# Nasal chondromesenchymal hamartoma in young children: CT and MRI findings and review of the literature

**DOI:** 10.1186/1477-7819-12-257

**Published:** 2014-08-12

**Authors:** Tingting Wang, Wenhua Li, Xiangru Wu, Qianqian Li, Yanfen Cui, Caiting Chu, Mingliang Xiang, Gang Ren

**Affiliations:** Department of Radiology, Xinhua Hospital affiliated to Shanghai Jiao Tong University School of Medicine, 1665 Kong Jiang Road, Shanghai, 200092 China; Department of Pathology, Xinhua Hospital affiliated to Shanghai Jiao Tong University School of Medicine, 1665 Kong Jiang Road, Shanghai, 200092 China; Department of Otolaryngology-Head & Neck Surgery, Xinhua Hospital affiliated to Shanghai Jiao Tong University School of Medicine, 1665 Kong Jiang Road, Shanghai, 200092 China

**Keywords:** Computerized tomography, Magnetic resonance imaging, Nasal chondromesenchymal hamartoma

## Abstract

**Background:**

Nasal chondromesenchymal hamartoma (NCMH) is an extremely rare benign tumor, primarily diagnosed in young infants and children and it often simulates malignant tumors on imaging.

**Case presentation:**

We present computerized tomography and magnetic resonance imaging findings of two nasal chondromesenchymal hamartomas in a 5-year-old boy and a 6-week-old girl.

**Conclusions:**

NCMH is a rare, benign tumor-like lesion with good biologic behavior. No recurrence after complete resection or malignant transformation of NCMH has been reported. A correct diagnosis is imperative to avoid unnecessary adjuvant therapy.

## Background

Nasal chondromesenchymal hamartoma (NCMH) is a rare, relatively newly discovered, pediatric tumor. In 1998, McDermott et al. [1] were the first to use the term NCMH to describe a tumor consisting mainly of chondroid or cartilaginous tissues and mesenchymal elements that occurred in the nasal cavity and had similar morphology to chest wall mesenchymal hamartoma occurring exclusively in infancy. Reported cases have occurred mostly in infants and young children, with characteristic histopathological features similar to chest wall mesenchymal hamartoma in infancy [1–5]. However, the affected age range can vary from newborn to the very elderly [1, 6–11].

To the best of our knowledge, NCMH has been reported in less than 25 cases in the English medical literature [11, 12]; thus, the pathogenesis of NCMH remains poorly understood. In this paper, we present computed tomography (CT) and magnetic resonance imaging (MRI) findings of NCMH in two young children.

## Case presentation

### Case 1

**Figure 1:**
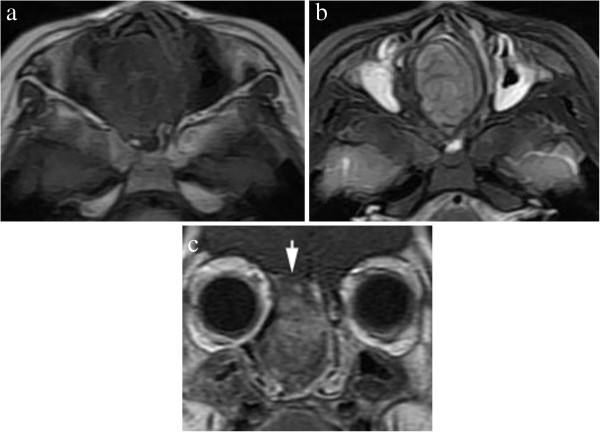
**CT of sinonasal cavity. (a)** An axial non-contrast CT revealed an ovoid soft-tissue mass in the right nasal cavity. **(b** **)** An axial contrast-enhanced CT image showed marked enhancement. **(c)** A coronal contrast-enhanced CT image revealed a marked enhanced mass which extends into the anterior cranial fossa and erodes the cribriform plate (arrow). The nasal septum was deviated to the left and the right nasal cavity dilated.

**Figure 2:**
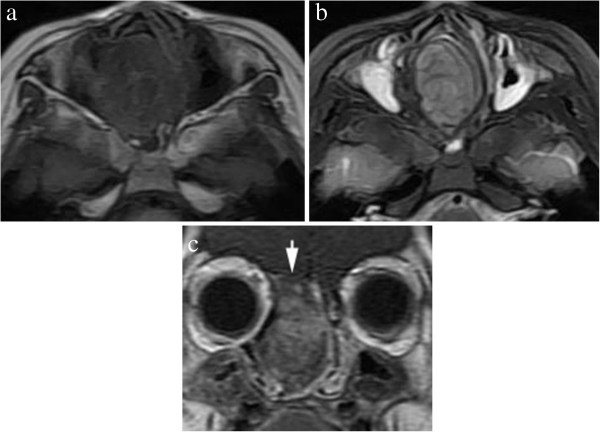
**MR Imaging of sinonasal cavity. (a)** An axial T1-weighted MRI revealed a mildly hypointense mass in the right nasal cavity. **(b)** An axial T2-weighted MRI showed a well-defined hyperintense mass and strong signal in both maxillary sinuses. **(c)** A coronal contrast-enhanced T1-weighted MRI showed striking enhancement of the mass, extending into the anterior fossa with intact dura.

**Figure 3:**
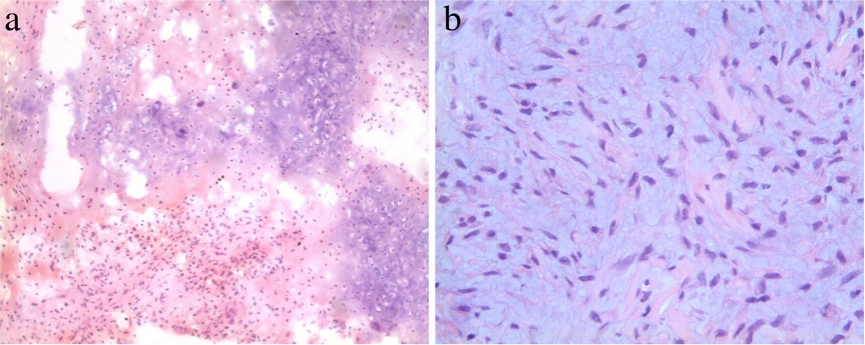
**High-power photomicrograph. (a)** A photomicrograph of the mass showing an irregular island of cartilage merged with spindle cells (hematoxylin-eosin, original magnification × 100). **(b)** A high-power photomicrograph showing spindle cells loosely arranged in myxoid stroma (hematoxylin-eosin, ×200).

A 5-year-old boy was admitted to our hospital for evaluation of nasal obstruction, recurrent sinusitis, and noisy breathing over a period of 4 years. The child had normal nutrition and development. Physical examination revealed a smooth, purple, non-tender mass attached to the roof of the right nasal cavity. No palpable cervical lymphadenopathy was identified. The remainder of his medical and family history was unremarkable. A CT scan revealed an oval-shaped, well-defined homogeneous soft-tissue mass in the right nasal cavity. The mass measured 2.5 × 3.6 × 4.3 cm and involved the ethmoid sinus, extending up to the cribriform plate and the anterior cranial fossa with evidence of bony erosion of the cribriform plate. Tumor density was measured in regions of interest in different slices and ranged from 33 to 36 Hounsfield Units (HU) before contrast injection; it was strikingly enhanced after injection, to a range of 64 to 69 HU (Figure 1). MRI demonstrated a well-demarcated homogeneous mass extending to the anterior cranial fossa in the right nasal cavity. The mass had low signal intensity on T1-weighted images and mixed high signal intensity on T2-weighted images, with striking inhomogeneous enhancement (Figure 2). No calcification, cystic component, or definite dural enhancement was identified. The right nasal cavity was dilated. The middle and inferior turbinates were medially compressed and the nasal septum was deviated to the left, but the mass did not invade adjacent structures. A biopsy was performed and the pathology report was consistent with NCMH. Operative findings revealed a well-defined mass attached to the anterior skull base, and a subtotal resection was performed. Histopathologically, the mass was composed mainly of irregular islands of chondroid tissues and mesenchymal elements such as spindle cells in a myxoid stroma (Figure 3). At present, a follow-up CT at 3 years revealed no recurrence.

### Case 2

**Figure 4:**
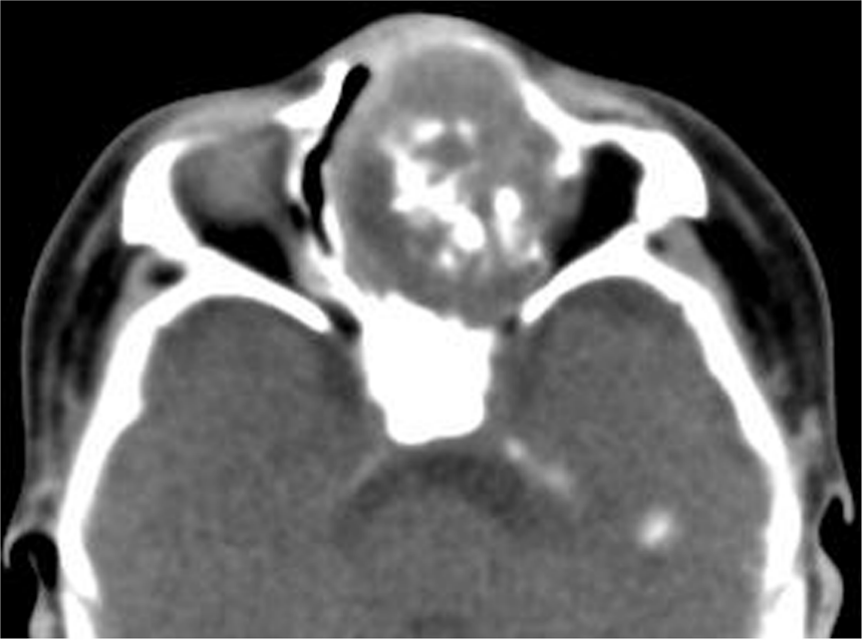
**An axial non-contrast CT revealed an well-defined, irregular, calcified soft-tissue mass in the left nasal cavity.**

**Figure 5:**
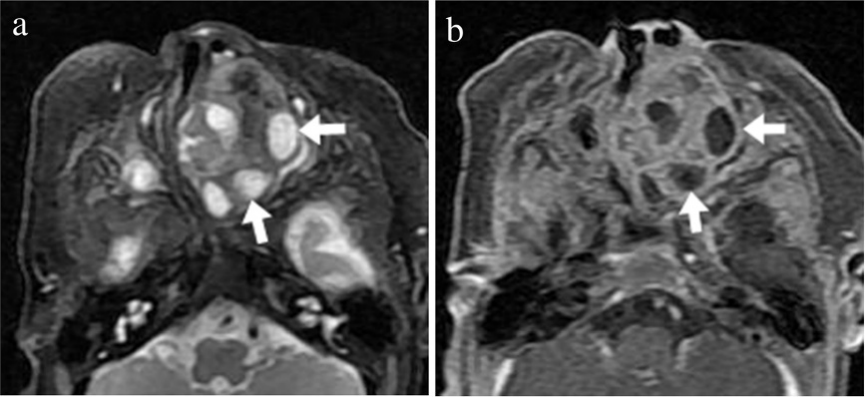
**MR Imaging of sinonasal cavity. (a)** An axial T2-weighted MRI showed a well-defined, heterogeneous hyperintense mass containing multiple small round areas of higher signal intensity (arrow). **(b)** Axial contrast-enhanced fat-suppressed T1-weighted image demonstrated striking heterogeneous enhancement of the mass with multiple non-enhanced cystic components within the lesion (arrow).

A 6-week-old girl, born at full term by transvaginal delivery, presented with a 4-week history of left nasal watery rhinorrhea and obstruction. Physical examination revealed a purple polypoid mass in the left nasal cavity. All other findings of her medical and family history were unremarkable. Non-contrast CT scans revealed a 2.6 × 3.4 × 3.9 cm well-defined, expansile mass with amorphous calcification in the left nasal cavity (Figure 4). The mass caused pressure remodeling of the adjacent bones without evidence of destruction or invasion of the adjacent structures. MRI demonstrated that the signal intensity of the mass was heterogeneous on T1- and T2-weighted images. The T2-weighted images further showed multiple round areas of high signal intensity within the lesion. The majority of the mass showed a strongly heterogeneous enhancement and the multiple round areas of high signal intensity on T2-weighted images were demonstrated as non-enhancing cystic components after administration of contrast medium (Figure 5). Total resection of the mass was performed in this patient. Histopathologically, the mass was composed of multiple irregular cartilage islands in mesenchymal elements such as spindle cells in a myxoid stroma. The patient is currently doing well postoperatively, without evidence of residue or recurrence according to a 10-month follow-up CT scan.

## Discussion

The most common sinonasal tumors are epithelial in nature. However, nasal masses, including those of epithelial and mesenchymal origin, are infrequently encountered in young infants and children. Nasal hamartomatous lesions may be predominantly composed of mesenchymal and epithelial elements. Several different types of hamartomatous lesions, including angiomatous, lipomatous, chondroid, neurogenic, and epithelial hamartomas, depending on preponderant tissue, have been documented in the literature [4–7]. Mesenchymal hamartomas are more common than epithelial lesions, but a hamartoma of purely epithelial elements has not been described in children [8].

The initial diagnosis of NCMH can be difficult or mistaken radiographically. A correct diagnosis is made primarily from histopathologic examination. However, CT or MRI are diagnostic imaging techniques of choice because they can reveal the tumor’s site of origin, extension, and relationships to adjacent structures, and can also help determine the texture of the mass and aid in planning resection surgery. Most reported cases involved a heterogeneous soft-tissue mass, which were predominantly solid and cystic with or without calcification on a CT scan [1, 3, 7, 10, 11]. The solid component of the tumor is usually strongly enhanced. The lesion is inhomogeneous with low signal intensity on T1-weighted images and with high signal intensity on T2-weighted images with strongly inhomogeneous contrast enhancement. Calcification may be an important diagnostic clue which may help differentiate it from other nasal masses. In a review of CT imaging characteristics of 15 reported cases with NCMH [11], 67% revealed bony remodeling, thinning, or erosion, 53% demonstrated ethmoid sinus or intracranial extension through the cribriform plate, 50% demonstrated internal calcifications, 40% showed cystic components, and 67% demonstrated moderate to strong enhancement. In our two cases, CT of the lesion showed a well-demarcated homogeneous or heterogeneous soft-tissue mass with strong enhancement. In addition, it had low signal intensity on T1-weighted MRI and high signal intensity on T2-weighted images, with marked heterogeneous enhancement; 50% demonstrated calcification or apparent cystic portions within the lesions, which is similar to other reported cases in the literature [11].

The differential diagnosis of NCMH mainly includes nasoethmoidal encephalocele, nasal glioma, rhabdomyosarcoma, lymphoma, and chondrosarcoma. Nasoethmoidal encephaloceles often show mixed density or central soft tissue and peripheral water without marked enhancement, as well as a defect in bone, but no destruction of the anterior cranial fossa [13]. Nasal gliomas embryologically connect to the brain and are partially or completely sealed off with the same signal characteristics as the brain on MRI [12, 13]. Nasal lymphomas are usually homogeneous soft-tissue masses with uniform mild enhancement that distinguish them from NCMH. It can be very difficult to differentiate NCMH from rhabdomyosarcoma and chondrosarcoma radiographically, but the latter two tend to have a much more destructive, ill-defined, and rapid-growing nature [14].

## Conclusions

We present two cases of NCMH to alert others that an intranasal mass should be added to the list of differential diagnoses. NCMH is a rare, benign tumor-like lesion with good biologic behavior. No recurrence after complete resection or malignant transformation of NCMH has been reported. A correct diagnosis is imperative to avoid unnecessary adjuvant therapy.

## Consent

Written informed consent was obtained from the patient for publication of this case report and any accompanying images. A copy of the written consent is available for review by the editor-in-chief of this journal.

## Abbreviations

CT: Computed tomography
HU: Hounsfield Units
MRI: Magnetic resonance imaging
NCMH: Nasal chondromesenchymal hamartoma.
